# Determinants and Stratification of Microvascular Complications of Type 2 Diabetes Mellitus

**DOI:** 10.7759/cureus.44348

**Published:** 2023-08-29

**Authors:** Priya Reddy Mallimala, Kavita Shah, Mohit Mirchandani, Karan Sharma

**Affiliations:** 1 Medicine, Kurnool Medical College, Bangalore, IND; 2 Medicine, North Carolina Medical Center, Benson, USA; 3 Internal Medicine, Charles University in Prague, Prague, CZE; 4 Medicine, Poonam Multispecialty Hospital, Ahmedabad, IND

**Keywords:** type 2 diabetes mellitus, diabetic nephropathy, diabetic neuropathy, diabetic retinopathy, kdigo

## Abstract

Background

Diabetes mellitus (DM) is a prevalent metabolic disorder characterized by high blood sugar levels. It is classified into type 1 (T1DM) and type 2 (T2DM), which have different mechanisms and complications. The global prevalence of diabetes, particularly T2DM, has increased significantly in recent decades, leading to a need for standardized data collection of macrovascular and microvascular complications to track disease progression and guide treatment options. This study aims to assess and correlate the prevalence and severity of microvascular complications in patients with T2DM.

Methodology

This observational, cross-sectional study was conducted at Poonam Multispeciality Hospital in Ahmedabad, India. A total of 4,123 diabetic patients admitted to the hospital were included. Information on sociodemographics and medical history was collected using standardized forms. Fundus photography and fluorescein angiography were performed to assess diabetic retinopathy, and estimated glomerular filtration rate and albumin-to-creatinine ratio were measured to evaluate renal function. Neurological examinations were conducted to score diabetic neuropathy. Chi-square tests were used to determine associations between medical history with diabetic retinopathy and nephropathy, and t-tests were used to compare diabetic neuropathy scores. Kendall’s Tau correlation was used to determine correlations between diabetic retinopathy and nephropathy. P-values <0.05 were considered statistically significant.

Results

The overall prevalence of diabetic retinopathy, nephropathy, and neuropathy was 37.5%. Of the patients included, 47.9% had diabetic nephropathy and 28.9% had diabetic neuropathy. A significant association was observed between the severity of diabetic retinopathy and age, body mass index, duration of diabetes, and hemoglobin A1c (HbA1C) levels. Similarly, significant associations were found between these factors and the severity of diabetic nephropathy. Unpaired t-tests revealed significant differences in diabetic neuropathy examination scores based on the duration of diabetes and Hba1C levels. Moreover, correlation analysis indicated a low, positive correlation between diabetic retinopathy and diabetic nephropathy.

Conclusions

This study provides insights into the prevalence, severity, and associations of microvascular complications in patients with T2DM, contributing to the understanding and management of these conditions. Additionally, the research revealed a direct association between diabetic retinopathy and different stages of chronic kidney disease determined by the Kidney Disease Improving Global Outcome guidelines.

## Introduction

Diabetes mellitus (DM) is a group of prevalent metabolic disorders that exhibit symptoms of high blood sugar levels. These disorders result from the interplay of various genetic and environmental factors. DM is categorized based on the pathogenic mechanism that triggers high blood sugar levels. The two primary classifications of DM are type 1 (T1DM) and type 2 (T2DM). T1DM arises due to autoimmunity against beta cells, which generate insulin, leading to severely reduced insulin levels. T2DM encompasses a range of disorders characterized by varying levels of insulin resistance, impaired insulin secretion, and heightened glucose production in the liver [1].

DM can affect multiple organs resulting in morbidity and eventual mortality. These complications are categorized as vascular and non-vascular in both types of diabetes. Vascular complications are further divided into macrovascular, such as coronary heart disease, stroke, and peripheral vascular disease, and microvascular, such as retinopathy, peripheral neuropathy, nephropathy, end-stage renal disease, and lower-extremity amputation.

In recent decades, the prevalence of diabetes has surged across many developed global regions; currently, approximately 415 million individuals globally are living with diabetes [2]. In 2000, the global occurrence of diabetes across all age ranges was approximately 2.8% and is expected to increase to approximately 4.4% by 2030. The projected number of individuals with diabetes is estimated to escalate from 171 million in 2000 to 366 million in 2030 [3]. With the growing prevalence of diabetes, data collection on complications needs to be further standardized not just in high-income countries but globally. The complication load associated with diabetes is largely due to macrovascular and microvascular complications.

Of the global population affected by DM, nearly half exhibit some form of diabetic retinopathy [4]. Systemic risk factors include duration of DM, type of DM, age, glycemic control, hypertension, renal disease, dyslipidemia, pregnancy, smoking, and alcohol consumption. Ocular risk factors include posterior vitreous detachment, cataract surgery, and chorioretinopathy. Duration of DM and level of glycemic control are the most robust indicators of diabetic retinopathy [5,6].

Diabetic nephropathy is a significant cause of chronic renal failure; approximately 20% of patients with T2DM develop end-stage renal disease. Diabetic nephropathy is a long-term complication that significantly impacts morbidity and mortality among diabetic patients. While the estimated glomerular filtration rate (eGFR) is the traditional marker for overall kidney function, albuminuria is a key indicator of damage to the glomerular permeability barrier [7,8]. The current classification of chronic kidney disease (CKD) based on Kidney Disease Improving Global Outcome (KDIGO) guidelines takes into account both eGFR and albuminuria [9]. This study aims to stratify renal risk using KDIGO classification and assess its association with the degree of diabetic retinopathy among individuals with T2DM.

The prevalence of diabetic peripheral neuropathy ranges from 7% within one year of diagnosis to 50% in individuals who have had diabetes for over 25 years. If individuals with subclinical neuropathic disturbances are included, the prevalence may exceed 90% [10]. Diabetic peripheral neuropathy initially affects the distal lower extremities. As the disease progresses, sensory loss ascends to the legs and appears in the hands, resulting in typical stocking and glove sensory loss.

This study aimed to determine the prevalence of diabetic retinopathy, nephropathy, and neuropathy in patients with DM, assess the association of various demographic features and medical history with the severity of these microvascular complications, and analyze the correlation between the severity of diabetic retinopathy and diabetic peripheral nephropathy.

## Materials and methods

This observational, cross-sectional study was conducted at Poonam Multispeciality Hospital. Ahmedabad, India, from April 2021 to April 2022, after obtaining approval from the institutional ethical committee (approval number: ECR/341/Inst/GJ/2012/RR-18). The study was conducted among patients admitted to the hospital’s medicine inpatient unit and intensive care unit. During the study period, a total of 4,123 patients with diabetes were admitted. Patients 18 years of age or above who consented to participate in the study were included. Patients younger than 18 years of age, those who did not consent to participate, pregnant females, and those unable to provide a medical history due to critical conditions were excluded. Thus, excluding the patients who met the exclusion criteria, a total of 3,000 patients were interviewed and included.

A standard pre-evaluation form was designed to obtain medical information from participants. It consisted of sociodemographic data, medical history, and personal history. Fundus photography and fluorescein angiography were performed by the ophthalmologist for each participant to assess diabetic retinopathy. Classification of diabetic retinopathy was based on the evidence-based approach of the Early Treatment Diabetic Retinopathy Study [11]. Retinopathy was classified as non-proliferative diabetic retinopathy (NPDR) or proliferative diabetic retinopathy (PDR). Patients were divided into the following groups based on diabetic retinopathy severity: mild NPDR, moderate NPDR, severe NPDR, early PDR, and high-risk PDR.

**Figure 1:**
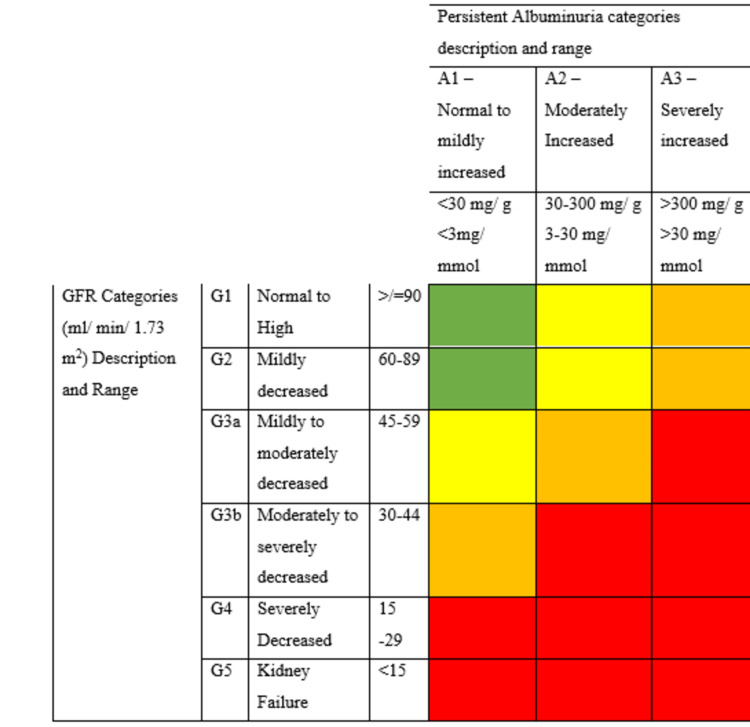
Division of patients and severity of diabetic nephropathy based on Kidney Disease Improving Global Outcome (KDIGO) classification. Rani et al. [13]. Permission to reproduce was obtained from the publisher. LR (low risk) includes, A1G1, A1G2; MR (moderate risk) includes A1G3a, A2G1, A2G2; HR (high risk) includes A1G3b, A2G3a, A3G1, A3G2; VHR (very high risk) includes A1G4, A1G5, A2G3b, A2G4, A2G5, A3G3a, A3G3b, A3G4, A3G5. Green: low risk (if no other markers of kidney disease, no chronic kidney disease); yellow: moderately increased risk; orange: high risk; red: very high risk.

eGFR and albumin-to-creatinine ratio were measured to assess renal function. Biochemical parameters such as hemoglobin A1c (HbA1c), creatinine, urinary protein, and urinary albumin were also recorded. eGFR was measured using the CKD-EPI equation [12]. Based on the study conducted by Rani et al., we divided our study population into low-risk (LR) (green), moderate-risk (MR) (yellow), high-risk (HR) (orange), and very high-risk (VHR) (red) according to the KDIGO classification, based on eGFR and urinary albumin values [13]. Figure 1 shows the categorization of subjects and severity of diabetic nephropathy based on the KDIGO classification.

To diagnose the presence of neuropathy and assess severity, a thorough neurological examination was performed for each participant, and neurological signs were scored based on the diabetic neuropathy examination (DNE). This test consists of eight items, which test muscle strength, tendon reflex, and sensations. The maximum score is 16, where >3 is considered abnormal [14]. Table 1 summarizes the DNE assessment scores [15].

**Table 1:** Diabetic neuropathy examination (DNE) assessment and scores.

DNE item		Description (score for item)
Muscle strength	Quadriceps femoris, extension of the knee	0 = normal; 1 = Medical Research Council Scale 3-4; 2 = Medical Research Council Scale 0-2
	Tibialis anterior, dorsiflexion of the foot	
Reflex	Triceps surae	0 = normal; 1 = mild/moderate deficit; decreased but present; 2 = absent
Sensation	Index finger: sensitivity to pinprick	0 = normal; 1 = decreased but present; 2 = absent
	Big toe: sensitivity to pinprick, sensitivity to touch, vibration perception, sensitivity to joint position	

Height, weight, and blood pressure were recorded by the investigator and entered into Google Forms. All data were exported to MS Excel (Microsoft Corp., Redmond, WA, USA) and analyzed using suitable tests for significance by SPSS version 26 (IBM Corp., Armonk, NY, USA) and MS Excel. Chi-square, analysis of variance (ANOVA), or t-tests were performed as applicable to compare variables between groups. P-values <0.05 were considered statistically significant. To measure the association of the ordinal variables (diabetic retinopathy severity against different study groups, i.e., LR, MR, HR, and VHR, based on KDIGO classification), Kendall’s tau-c correlation coefficient test was used to determine the probability of concordance minus the probability of discordance pairs. The data were analyzed using SPSS version 21 and stratified based on confounding factors (medication regimen, age, duration, and severity of diabetes).

## Results

**Figure 2:**
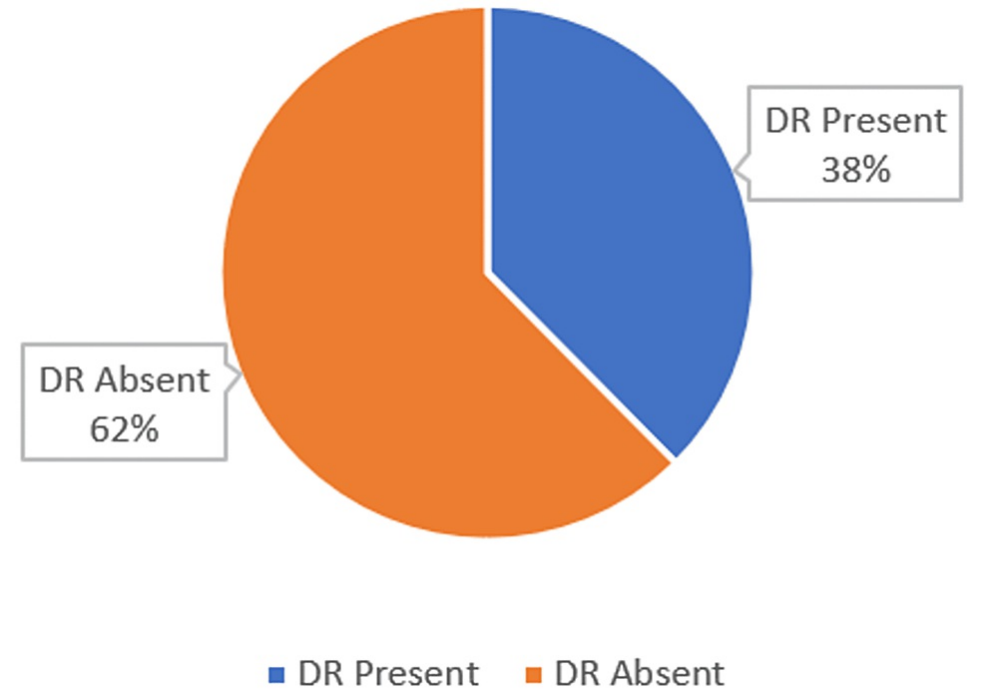
Prevalence of diabetic retinopathy. DR: diabetic retinopathy

**Figure 3:**
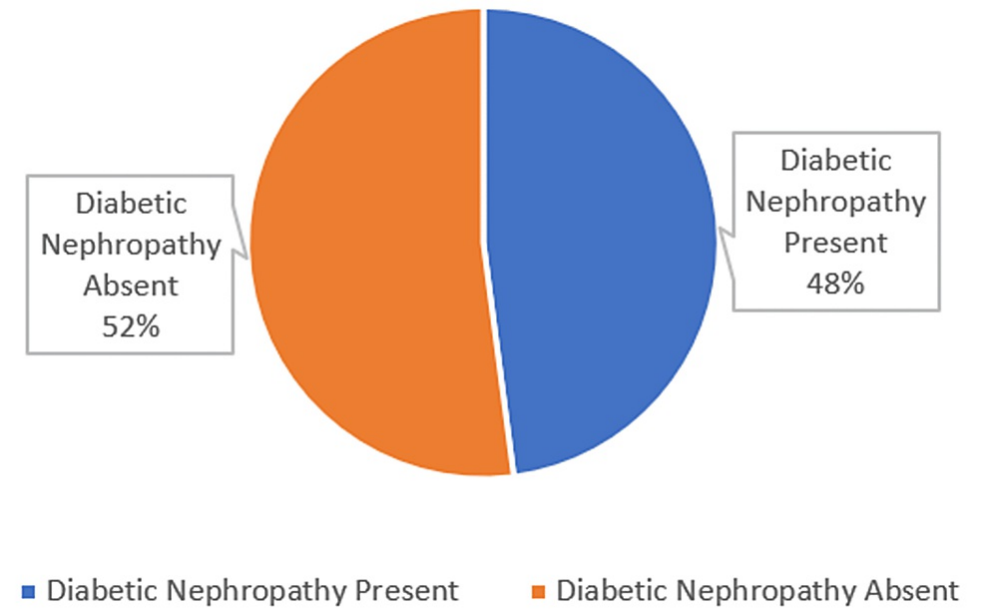
Prevalence of diabetic nephropathy.

**Figure 4:**
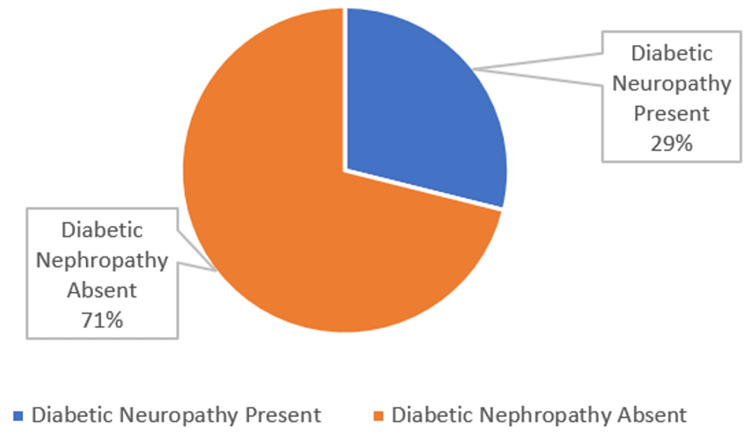
Prevalence of diabetic neuropathy.

Of the 3,000 participants, 1,127 (37.5%) were diagnosed with diabetic retinopathy, 1,438 (47.9%) were diagnosed with diabetic nephropathy, and 867 (28.9%) had diabetic neuropathy (Figures 2-4).

The chi-square analysis showed a significant association between the severity of diabetic retinopathy and age, body mass index (BMI), duration of DM, and HbA1C (p < 0.05) (Table 2).

**Table 2:** Association of severity of diabetic retinopathy with patient medical history. BMI: body mass index; DM: diabetes mellitus; HbA1C: hemoglobin A1C; NPDR: non-proliferative diabetic retinopathy; PDR: proliferative diabetic retinopathy

		Severity of diabetic retinopathy					Chi-square	P-value
		Mild NPDR	Moderate NPDR	Severe NPDR	Early PDR	High-risk PDR		
Sex	Male	154	136	112	125	43	0.24	0.99
	Female	156	132	106	119	44	177.81	<0.05
Age (years)	25–35	3	14	21	4	0		
	36–45	56	34	53	21	7		
	46–55	88	51	94	73	12		
	56–65	213	125	66	39	78		
	66–75	8	12	2	6	3		
	76–85	16	7	3	14	4		
BMI (kg/m^2^)	<18.5	23	11	8	4	1	36.99	<0.05
	18.5–24.9	133	37	31	22	15		
	25.0–29.9	248	127	99	32	21		
	≥30	146	102	46	8	13		
DM duration (years)	≤5	152	34	23	3	0	368	<0.05
	6–10	39	54	17	7	5		
	11–15	21	43	11	31	3		
	16–20	116	58	41	77	19		
	21–25	89	108	125	2	0		
	26–30	14	12	16	5	2		
HbA1C (%)	6.5–7.0	56	17	8	25	28	139.5	<0.05
	7.1–7.5	45	55	70	32	74		
	7.6–8.0	85	35	71	101	60		
	8.1–8.5	55	20	32	48	64		
	8.6–9.0	8	16	10	31	42		
	9.1–9.5	3	5	8	2	4		
	9.6–10	2	1	4	1	3		
	≥10	2	2	1	1	0		
Blood pressure (mmHg)	Normal <120/<80 mmHg	42	36	18	24	36	15.58	0.21
	Pre-hypertension 120–139/80–89 mmHg	72	90	48	75	64		
	Stage I hypertension 140–159/90–99 mmHg	123	132	98	118	122		
	Stage II hypertension ≥160/≥100	4	5	9	6	5		

The chi-square analysis showed a significant association between the severity of diabetic nephropathy and age, BMI, duration of diabetes mellitus, and HbA1C (p < 0.05) (Table 3).

**Table 3:** Association of severity of diabetic nephropathy with patient medical history. BMI: body mass index; DM: diabetes mellitus; HbA1C: hemoglobin A1C; KDIGO: Kidney Disease Improving Global Outcome; LR: low risk; MR: moderate risk; HR: high risk; VHR: very high risk

		The severity of diabetic nephropathy (KDIGO)				Chi-square	P-value
		LR	MR	HR	VHR		
Sex	Male	182	305	101	158	7.606	0.054
	Female	189	305	88	110		
Age (years)	25–35	23	65	42	26	96.84	0.00
	36–45	87	124	45	57		
	46–55	122	89	128	50		
	56–65	142	97	122	67		
	66–75	22	45	18	22		
	76–85	12	10	8	15		
BMI (kg/m^2^)	<18.5	14	18	7	17	116.4	0.00
	18.5–24.9	101	143	89	79		
	25.0–29.9	128	245	72	94		
	≥30	127	98	154	52		
DM duration (years)	≤5	10	9	12	13	68.12	<0.001
	6–10	125	93	77	86		
	11–15	35	81	48	55		
	16–20	95	114	93	96		
	21–25	53	47	79	27		
	26–30	56	34	62	38		
HbA1C (%)	6.5–7.0	79	63	53	46	59.53	0.00
	7.1–7.5	61	47	77	48		
	7.6–8.0	68	93	45	52		
	8.1–8.5	80	123	66	68		
	8.6–9.0	30	27	28	27		
	9.1–9.5	48	57	68	28		
	9.6–10	11	13	8	14		
	≥10	5	3	2	0		
Blood pressure (mmHg)	Normal <120/<80 mmHg	89	128	74	83	58.73	0.07
	Pre-Hypertension 120–139/80–89 mmHg	142	135	111	124		
	Stage I hypertension 140–159/90–99 mmHg	58	76	89	23		
	Stage II hypertension ≥160/≥100	91	59	93	63		

An independent t-test was performed to compare the mean DNE scores between males and females. One-way ANOVAs were performed to compare the means of the remaining parameters. A significant difference was observed between the mean DNE score based on the duration of DM (p = 0.03) and HbA1C (p < 0.001) (Table 4).

**Table 4:** Comparison of diabetic neuropathy examination mean scores. BMI: body mass index; DM: diabetes mellitus; HbA1C: hemoglobin A1C

		n	Mean score		P-value
Sex	Male	524	9.8 ± 3.75	t = 0.52	0.6
	Female	343	9.67 ± 3.69		
Age (years)	25–35	42	10.1 ±3.96	F = 0.66	0.652
	36–45	65	10.4 ± 3.65		
	46–55	144	10.22 ± 3.42		
	56–65	253	10.07 ± 3.72		
	66–75	184	10.08 ± 3.91		
	76–85	177	10.11 ± 3.83		
BMI (kg/m^2^)	<18.5	106	9.74 ± 4	Df (3, 318) F = 0.55	0.74
	18.5–24.9	345	9.9 ± 3.65		
	25.0–29.9	212	10.01 ± 3.71		
	≥30	204	10.22 ± 3.8		
DM duration (years)	≤5	111	10.12 ± 3.85	Df (5,425) F = 2.51 Between 21–25 and 26–30	0.03
	6–10	192	9.65 ± 3.74		
	11–15	147	10.08 ± 3.74		
	16–20	203	9.59 ± 3.67		
	21–25	85	10.22 ± 3.86		
	26–30	129	10.27 ± 3.79		
HbA1C (%)	6.5–7.0	78	9.9 ± 3.88	Df (7,308) F = 5.04	<0.001
	7.1–7.5	98	10.71 ± 3.85		
	7.6–8.0	149	9.79 ± 3.98		
	8.1–8.5	175	10.21 ± 3.81		
	8.6–9.0	176	9.91 ± 3.68		
	9.1–9.5	84	10.77 ± 3.52		
	9.6–10	65	11.57 ± 3.43		
	≥10	42	13.34 ± 3.11		
Blood pressure (mmHg)	Normal <120/<80 mmHg	221	10.04 ± 3.79	F = 2.57	0.082
	Pre-hypertension 120–139/80–89 mmHg	307	10.26 ± 3.76		
	Stage I hypertension 140–159/90–99 mmHg	331	10.32 ± 3.79		
	Stage II hypertension ≥160/≥100	8	11.88 ± 2.23		

Kendall’s Tau correlation was performed to determine the correlation between diabetic retinopathy and diabetic nephropathy. We found a low, positive correlation between these parameters (r = 0.16, p = 0.001) (Table 5).

**Table 5:** Correlation between diabetic retinopathy and diabetic nephropathy Kendall’s Tau correlation coefficient (r) =  0.16, p < 0.001 (for all the cases). NPDR: non-proliferative diabetic retinopathy; PDR: proliferative diabetic retinopathy; LR: low risk; MR: moderate risk; HR: high risk; VHR: very high risk

Variables	LR (number of participants)	MR (number of participants)	HR (number of participants)	VHR (number of participants)
Mild NPDR	25	27	29	30
Moderate NPDR	29	31	33	38
Severe NPDR	43	45	47	48
Early PDR	33	38	34	42
High risk PDR	27	29	31	36

## Discussion

Prevalence of microvascular complications

This study reported a prevalence of 37.5% of diabetic retinopathy, 47.9% of diabetic nephropathy, and 28.9% of diabetic neuropathy. Ramachandran et al. conducted a study in 1999 and showed that the prevalence of diabetic retinopathy was 23.7% and the prevalence of peripheral neuropathy was 27.5% [16]. A study conducted in India by Agrawal et al. in 2014 showed that retinopathy diagnosis was made in 32.5% of cases, while nephropathy was present in 30.2% and peripheral neuropathy in 26.8% of cases [17]. Remal et al. conducted a study in 1996 and reported that the prevalence of retinopathy was 34.1% in T2DM [18]. The variation in the prevalence of diabetic nephropathy across different geographical locations and populations may be attributed to ethnic differences in susceptibility to the condition, such as genetic factors. Additionally, poor management of diabetes, hypertension, and various socioeconomic, cultural, and environmental factors can contribute to this variation. Furthermore, the quality and quantity of protein intake may also play a significant role in the development and progression of diabetic nephropathy.

Diabetic retinopathy

This study showed a significant association of diabetic retinopathy with age, BMI, duration of DM, and HbA1c levels. The study conducted by Pang et al. in 2012 showed that factors linked to retinopathy in diabetes included the duration of diabetes, blood glucose levels, HbA1C, and albuminuria [19]. Ahmed et al. conducted a study on factors associated with diabetic retinopathy in T2DM patients and showed that the presence of retinopathy exhibited significant associations with advanced age, early onset at a younger age, prolonged disease duration, inadequately managed blood sugar, hypertension, and insulin utilization. Notably, the presence of neuropathy and nephropathy emerged as significant risk factors. Among these, early onset at a younger age, longer disease duration, and insulin usage demonstrated the strongest predictive capability for diabetic retinopathy [20]. Raman et al. conducted a study in South India in 2014 which showed that the duration of T2DM was the strongest risk factor for diabetic retinopathy [21]. All of these studies have reported similar findings to our study.

Diabetic nephropathy

This study showed a significant association of nephropathy with age, BMI, duration of DM, and HbA1C levels. Ayodele et al. published a study in 2004 and reported that race, genetic susceptibility, hypertension, hyperglycemia, hyperfiltration, smoking, advanced age, male sex, and a high-protein diet were the factors associated with diabetic nephropathy [7]. The study conducted by Unnikrishnan et al. in India in 2007 showed that HbA1C, duration of DM, and systolic blood pressure were associated with overt nephropathy, which is consistent with our study findings [22].

Diabetic neuropathy

We used the DNE score to compare the means between different groups. A significant difference was observed between the mean DNE score based on the duration of DM and HbA1c. No difference was seen in the mean scores when gender, age, and blood pressure were compared. This indicates that the severity of diabetic neuropathy does not depend on gender, age, and blood pressure. However, more studies are needed to validate this score and compare it with more causal factors. According to a study conducted by Papanas and Ziegler in 2015, the most prevalent neurological symptom in diabetes is distal symmetric sensorimotor polyneuropathy (DSPN), which is primarily influenced by factors such as the duration of DM, high blood sugar levels, and age. Additionally, prediabetes, hypertension, dyslipidemia, and obesity are significant risk factors for developing DSPN [23]. A study by Pradeepa et al. in 2008 also showed that diabetic neuropathy was associated with age, HbA1C, and duration of DM [24].

Correlation between diabetic retinopathy and diabetic neuropathy

Our study showed a low, positive correlation between diabetic retinopathy and diabetic nephropathy. This finding was consistent with the study conducted by Anitharani et al. [13]. In a study conducted by Kotlarsky et al., a statistically significant relationship was observed between diabetic retinopathy and diabetic neuropathy, displaying a unidirectional correlation attributed to the chronological order in which diabetic neuropathy occurs before diabetic retinopathy. The findings of this study suggest that the extent of renal impairment is directly proportional to the severity of eye damage. Additionally, this association possesses a chronological dimension, with renal injury preceding retinal damage [25].

The current study relied on the KDIGO classification to derive its findings. Moreover, unlike other studies, the CKD-EPI formula was employed to calculate eGFR. Consequently, the results emphasized a notable connection between various levels of diabetic retinopathy and the severity of CKD based on the KDIGO classification. Furthermore, a decrease in eGFR was associated with the different stages of diabetic retinopathy. The primary strength of the study lies in its ability to compare various levels of CKD using the KDIGO classification, which incorporates both eGFR and albumin levels.

The major limitation of the study was the inability to follow up to note the progress of diabetic complications. Conducting a prospective longitudinal study that focuses on comprehensive interventions, considering the risk factors and their association with progression from moderate to higher risk categories, is advisable. Furthermore, more factors should be included in future studies ranging from other laboratory parameters to personal habits and environmental causes to better understand the risk factors and the progression of the complications.

## Conclusions

This cross-sectional study conducted in a western Indian population with T2DM emphasized the importance of stratifying renal risk using the KDIGO classification rather than solely relying on eGFR or albuminuria. Moreover, the study uncovered a positive correlation between diabetic retinopathy and the various stages of CKD based on the KDIGO classification.

## References

[1] Goyal R, Singhal M, Jialal I (2023). Type 2 Diabetes. https://www.ncbi.nlm.nih.gov/books/NBK513253/.

[2] Harding JL, Pavkov ME, Magliano DJ, Shaw JE, Gregg EW (2019). Global trends in diabetes complications: a review of current evidence. Diabetologia.

[3] Wild S, Roglic G, Green A, Sicree R, King H (2004). Global prevalence of diabetes: estimates for the year 2000 and projections for 2030. Diabetes Care.

[4] Ogurtsova K, da Rocha Fernandes JD, Huang Y (2017). IDF Diabetes Atlas: global estimates for the prevalence of diabetes for 2015 and 2040. Diabetes Res Clin Pract.

[5] Antonetti DA, Klein R, Gardner TW (2012). Diabetic retinopathy. N Engl J Med.

[6] West KM, Erdreich LJ, Stober JA (1980). A detailed study of risk factors for retinopathy and nephropathy in diabetes. Diabetes.

[7] Ayodele OE, Alebiosu CO, Salako BL (2004). Diabetic nephropathy--a review of the natural history, burden, risk factors and treatment. J Natl Med Assoc.

[8] Hemmelgarn BR, Manns BJ, Lloyd A (2010). Relation between kidney function, proteinuria, and adverse outcomes. JAMA.

[9] Lamb EJ, Levey AS, Stevens PE (2013). The Kidney Disease Improving Global Outcomes (KDIGO) guideline update for chronic kidney disease: evolution not revolution. Clin Chem.

[10] Yagihashi S, Mizukami H, Sugimoto K (2011). Mechanism of diabetic neuropathy: where are we now and where to go?. J Diabetes Investig.

[11] Wilkinson CP, Ferris FL 3rd, Klein RE (2003). Proposed international clinical diabetic retinopathy and diabetic macular edema disease severity scales. Ophthalmology.

[12] Levey AS, Stevens LA, Schmid CH (2009). A new equation to estimate glomerular filtration rate. Ann Intern Med.

[13] Anitha Rani A Kumpatla S, Viswanathan V (2017). Stratifying renal risk and retinal involvement in South Indian type 2 diabetic patients: using the KDIGO classification. Int J Diabetol Vasc Dis Res.

[14] Mythili A, Kumar KD, Subrahmanyam KA, Venkateswarlu K, Butchi RG (2010). A Comparative study of examination scores and quantitative sensory testing in diagnosis of diabetic polyneuropathy. Int J Diabetes Dev Ctries.

[15] Yang Z, Chen R, Zhang Y (2018). Scoring systems to screen for diabetic peripheral neuropathy. Cochrane Database Syst Rev.

[16] Ramachandran A, Snehalatha C, Satyavani K, Latha E, Sasikala R, Vijay V (1999). Prevalence of vascular complications and their risk factors in type 2 diabetes. J Assoc Physicians India.

[17] Agrawal RP, Ola V, Bishnoi P, Gothwal S, Sirohi P, Agrawal R (2014). Prevalence of micro and macrovascular complications and their risk factors in type-2 diabetes mellitus. J Assoc Physicians India.

[18] Rema M, Ponnaiya M, Mohan V (1996). Prevalence of retinopathy in non insulin dependent diabetes mellitus at a diabetes centre in southern India. Diabetes Res Clin Pract.

[19] Pang C, Jia L, Jiang S (2012). Determination of diabetic retinopathy prevalence and associated risk factors in Chinese diabetic and pre-diabetic subjects: Shanghai diabetic complications study. Diabetes Metab Res Rev.

[20] Ahmed RA, Khalil SN, Al-Qahtani MA (2016). Diabetic retinopathy and the associated risk factors in diabetes type 2 patients in Abha, Saudi Arabia. J Family Community Med.

[21] Raman R, Ganesan S, Pal SS, Kulothungan V, Sharma T (2014). Prevalence and risk factors for diabetic retinopathy in rural India. Sankara Nethralaya Diabetic Retinopathy Epidemiology and Molecular Genetic Study III (SN-DREAMS III), report no 2. BMJ Open Diabetes Res Care.

[22] Unnikrishnan RI, Rema M, Pradeepa R, Deepa M, Shanthirani CS, Deepa R, Mohan V (2007). Prevalence and risk factors of diabetic nephropathy in an urban South Indian population: the Chennai Urban Rural Epidemiology Study (CURES 45). Diabetes Care.

[23] Papanas N, Ziegler D (2015). Risk factors and comorbidities in diabetic neuropathy: an update 2015. Rev Diabet Stud.

[24] Pradeepa R, Rema M, Vignesh J, Deepa M, Deepa R, Mohan V (2008). Prevalence and risk factors for diabetic neuropathy in an urban south Indian population: the Chennai Urban Rural Epidemiology Study (CURES-55). Diabet Med.

[25] Kotlarsky P, Bolotin A, Dorfman K, Knyazer B, Lifshitz T, Levy J (2015). Link between retinopathy and nephropathy caused by complications of diabetes mellitus type 2. Int Ophthalmol.

